# On the importance of trip destination for modelling individual human mobility patterns

**DOI:** 10.1098/rsif.2020.0673

**Published:** 2020-10-14

**Authors:** Maxime Lenormand, Juan Murillo Arias, Maxi San Miguel, José J. Ramasco

**Affiliations:** 1 TETIS, Univ Montpellier, AgroParisTech, Cirad, CNRS, INRAE, Montpellier, France; 2 BBVA Data & Analytics, Avenida de Burgos 16D, 28036 Madrid, Spain; 3 Instituto de Física Interdisciplinar y Sistemas Complejos IFISC (CSIC-UIB), Campus UIB, 07122 Palma de Mallorca, Spain

**Keywords:** human mobility, complex systems, shopping trips

## Abstract

Obtaining insights into human mobility patterns and being able to reproduce them accurately is of the utmost importance in a wide range of applications from public health, to transport and urban planning. Still the relationship between the effort individuals will invest in a trip and the importance of its purpose is not taken into account in individual mobility models that can be found in the recent literature. Here, we address this issue by introducing a model hypothesizing a relation between the importance of a trip and the distance travelled. In most practical cases, quantifying such importance is undoable. We overcome this difficulty by focusing on shopping trips (for which we have empirical data) and by taking the price of items as a proxy. Our model is able to reproduce the long-tailed distribution in travel distances empirically observed and to explain the scaling relationship between distance travelled and item value found in the data.

## 1. Introduction

Individual human mobility is a complex phenomenon, involving various mechanisms interacting at different spatial and temporal scales. These dynamics are the product of individual behaviours, governed by decisions that may depend on multiple contextual factors such as economic resources, geography, culture, norms, habits or life experiences. However, beneath this apparent complexity lies remarkable temporal and spatial regularities in the way people travel and interact with their environment [1]. Results obtained in several studies based on dollar-bill tracking [2], mobile phone data [3], Twitter data [4,5], Foursquare data [6] and GPS data [7] suggest that the distance Δ_*r*_ between two consecutive locations follows a heavy-tailed distribution well approximated by a Pareto function $P(\Delta_{r})∼\Delta_{r}^{-(1+\alpha )}$ with 0 < *α* ≤ 1. It has also been shown that individuals tend to be attracted to popular places [8,9] and to return to previously visited locations, thus increasing the predictability of individual human movements [10] and allowing the identification of most visited locations as well as the characterization of daily commuting patterns [11]. Individual human mobility patterns are also strongly influenced by geographical constraints [12] but also by individuals’ socio-economic status [13–15] and social network [16–19].

Based on these empirical observations, several models have been proposed for modelling individual human mobility patterns. The simplest type describes human travelling using Lévy flights and continuous time random walks [2,20]. These models give accurate predictions but fail to reproduce some features such as the individuals’ tendency to revisit locations [3,20,21]. In [21], the authors propose a new model considering two generic mechanisms: exploration and preferential return, to decide whether an individual will visit a new place or a previously visited one as his/her next displacement. Going further in this direction, several models have been proposed to take into account diverse contextual factors such as the social context, urban geography and/or type and popularity of locations [9,11,12,22].

Nonetheless, most of these models focus on stationary (long-term) mobility, and, most importantly, they do not take into account the characteristics of the destination such as the purpose of travel and its importance to the individual. Indeed, one can assume that an individual will invest time or money, more generically, effort or amount of ‘energy’ into a trip according to the value attached to the destination/objective of this travel. The purpose of a basic trip is the displacement between home and work, the details of which have been collected in censuses for decades (in the USA, for instance, since 1990). The introduction of new GPS-based technologies have enabled the exploration of the purpose of other trips since the early 2000s [23,24]. Even though the relationship between trip cost and destination importance has been postulated in transport economy, and more recently in ecology, with the use of travel cost methods to assess the value of a natural sites based on the time and travel cost expenses that people spent to visit this site [25,26], without adequate empirical datasets to explicitly assess the ‘value’ of a destination this feature is rarely modelled at an individual scale.

The purpose of this work is to understand the displacement distribution generated by a process in which the trips have a clear purpose and, therefore, an associated objective or subjective value *v*. The main assumption, straightforwardly checked in the data, is that the trips’ length, *d*, tends to increase with *v*. We start by presenting a shopping mobility dataset that we use in the analysis and in which we can assign an objective meaning to *v* as the price of the purchased items. This dataset contains information on bank card transactions made in the provinces of Barcelona and Madrid. Inspired by search processes for wild food resources in the natural environment [7,27–29], we introduce a human individual mobility model taking into account the value given to the trip's destination through a parameter *p*, accounting for the probability of stopping or satisfying a search and that decreases when the value of *v* increases. The model generates trip length distributions that mimic the empirical ones and it is able to explain the observed scaling relations from the data.

## 2. Material and methods

### 2.1. Data

To explore the relationship between travel cost *d* and the importance given to its destination *v*, we analyse a credit card dataset containing information about 35 million bank card transactions made by card holders (hereafter called users) of the Banco Bilbao Vizcaya Argentaria (BBVA) in the province of Barcelona and Madrid in 2011. Each transaction is characterized by its amount (in euro currency) and a timestamp. Each transaction is also linked to a user and a business using anonymized user and business IDs. Users are identified with an anonymized user ID and their postcode of residence. In the same way, businesses are identified with an anonymized business ID, a business category (accommodation, automotive industry, bars and restaurants, etc.) and the geographical coordinates of the credit card terminal (see electronic supplementary material, table S2 for a full list of the selected business categories). The mobility habits and the representativeness of the BBVA credit card users in Barcelona and Madrid have already been investigated in [14,30]. Here, we filtered out users with an average number of transactions per day higher than three (see the electronic supplementary material for more details). Only credit card payments whose amount was inferior to 500 euros have been considered. Table 1 presents the final number of users, businesses and transactions analysed in this study.

**Table 1.** Number of users, businesses and transactions in both case studies. The number of postcodes and inhabitants and the surface area of the two provinces are also displayed.

statistics	Barcelona	Madrid
number of postcodes	364	268
number of inhabitants	5 540 925	6 489 680
area (km^2^)	7733	8022
number of users	269 849	528 719
number of businesses	111 267	108 936
number of transactions	12 993 179	24 507 586

**Figure 1.**
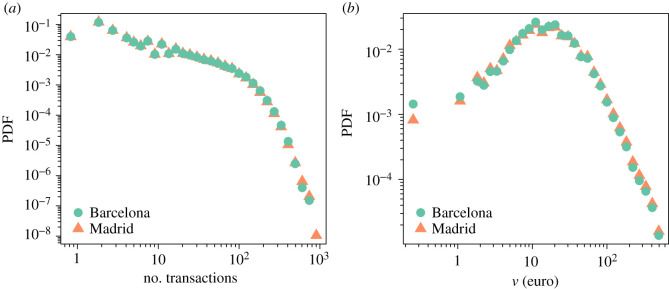
Probability density function of the number of transactions per user in 2011 (*a*) and the amount of money spent per transaction (*b*) in Barcelona (green dots) and Madrid (orange triangles).

The probability density function (PDF) of the number of transactions per user in 2011 and the amount of money spent per transaction is displayed in figure 1. We observe a strong heterogeneity among users regarding the number of transactions. The median value is 27 and the lower quartile is 8 in both provinces, the upper quartile is 69 for Barcelona and 66 for Madrid. Between 10 and 70 euros are spent in 50% of the transactions with a median amount of 30 euros per transaction.

For each transaction, the cost *d* associated with a trip is estimated with the distance between the user’s postcode of residence (lon/lat coordinates of the centroid) and the location of the business in which the transaction occurred (lon/lat of the credit card terminal). The value *v* given to the travel purpose is inferred by the amount of money spent per transaction. The amount of money spent *v* is divided into five intervals ([0, 50], [50, 75], [75, 100], [100, 200], [200, 500]).

### 2.2. Model

**Figure 2.**
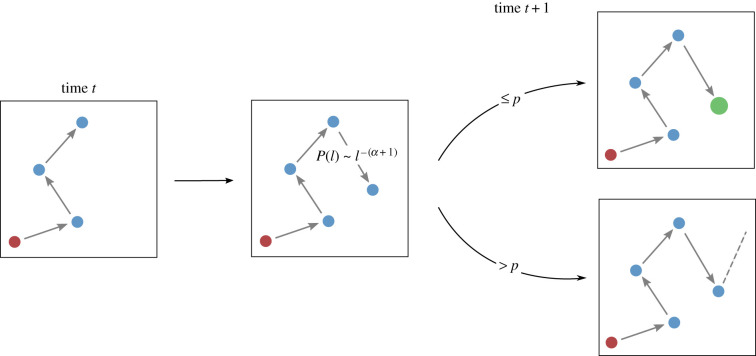
A schematic diagram of the model. At each step, the individual leaves his/her actual location and moves in a random direction at a distance sample from a Pareto distribution $P(l)=\alpha {l_{0}}^{\alpha }/l^{\alpha +1}$. If the new location falls outside of the square boundaries the sampling process is repeated. According to the value *v* given to the trip destination, the individual will then decide to stop or continue his/her journey with a probability *p*. If the individual decides to end his/her journey the final destination is drawn at random in a circle of radius *r* around the last position (green circle).

The proposed model can be interpreted as a search process that stops when a satisfying object (destination) has been found [31]. The rules of the model are outlined in figure 2. We assume that an individual starts the travel at his/her actual location (at home or work, for instance). The position of this first location is drawn at random in a square of lateral size *L* expressed in kilometres. In practice, this parameter takes into consideration the geographical constraints of a case study site (administrative or geological boundaries for example). At each step, the individual moves in a random direction and at a random distance sampled from a Pareto distribution $P(l)=\alpha {l_{0}}^{\alpha }l^{-\alpha -1}$, where *α* is the exponent and *l*_0_ the minimum spatial scale considered. At each step, the possibility of ending the trip is represented by a probability *p* of fulfilling the trip's goal. Note that unlike most of the models described in the introduction, since only short-range mobility patterns are considered, our model does not take time into account explicitly. The probability of stopping *p* is assumed to have an inverse relation with the importance given to the trip's goal *v*. The higher the value *v* associated with the objective of the travel, the longer the search process (i.e. low value of *p*) and the higher the distance *d* between origin and destination can become. If the purpose of the trip is a search to buy an object, the individuals would be willing to explore more shops or to travel further as the item price increases (buying a car requires more ‘energy’ than a piece of bread). Finally, when the individual decides to end his/her journey the final destination is drawn at random in a circle of radius *r* around the last position. This mechanism is included to take into account the uncertainty present in the data on the exact position of the retailing centre. In the case of a more abstract framework, the model could be simplified by making *r* → 0 and setting the purchase place in the current agent’s location.

### 2.3. Model calibration

The comparison between model and data is based on the PDF of the distance *d* between the origin and the destination. The simulated PDF is obtained with 100 000 simulations of the model. The model has five parameters *L*, *α*, *l*_0_, *r* and *p*. *L* controls the size of the modelling area, *l*_0_ and *r* the minimum spatial resolution, *α* the jump length and *p* the users’ tendency to explore the modelling area. The free parameter *l*_0_ and *r* allows notably to control the user's exploration behaviour at a short distance from home. The numerical values of these five parameters were determined from the empirical data in two steps and independently for both provinces. We first adjusted the values of the five parameters by minimizing the Kolmogorov–Smirnov distance between observed and simulated PDFs of *d* (all values of *v* combined). We then adjusted the value of *p* according to the PDF of *d* for each interval of amount of money spent *v*.

### 2.4. Model features

Every agent in the model is performing a short Lévy flight in the limited space provided by a box of side *L*. To better understand the model features, it is helpful to take first the limits *L* → ∞, so there is no spatial constraint. The Lévy flight is not, actually, complete because we introduce a stopping mechanism with *p*. It implies that the number of jumps, *n*, that an agent takes follows the geometric distribution:

2.1 $$P_{n}(n)={\left(1-p\right)}^{n-1}p.$$

**Figure 3.**
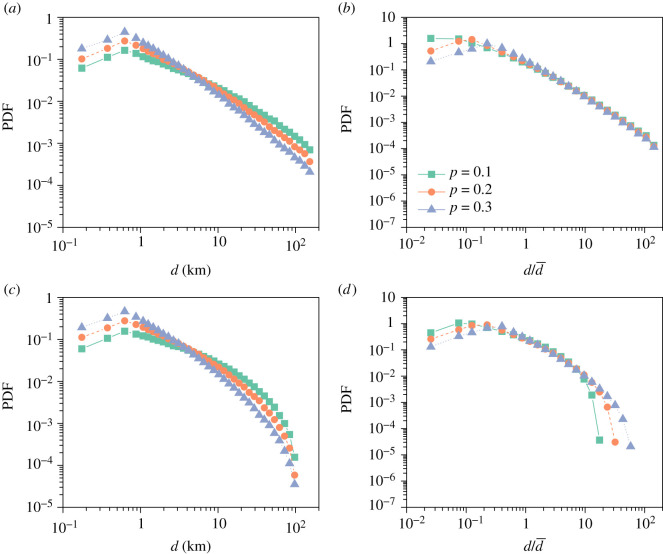
Probability density function of the distance *d* from the residence and the final location as a function of *p* obtained with the calibrated model for Barcelona. The original distributions are shown in (*a*) and (*c*), while the normalized distributions obtained by dividing by the median distance $\bar{d}$ are in (*b*) and (*e*). In (*a*) and (*b*), there is no bounding box, *L* = ∞, while in (*c*) and (*d*) *L* = 100 km.

The average number of jumps is thus given by 〈*n*〉 = 1/*p*. Lower *p* means more jumps and, therefore, the potential for longer distances in the distribution of distance from the origin to the purchase location, *P*(*d*). Recalling that *p* will be related to *v* with an inverse function, larger *v* also implies longer distances *d*. The distribution *P*(*d*) comes thus from the aggregation of a finite number *n* of Lévy jumps. In the limit *n* → ∞, it could be expressed as a function of the Lévy *α*-stable distributions. On the contrary, for small *n* the analytical expression of *P*(*d*) does not correspond to Lévy’s generalization of the central limit theorem. In any case, there is an inverse relation between the median of the distance *d*, $\bar{d}$ and *p*. Examples of the distributions *P*(*d*) for different values of *p* can be seen in figure 3*a*. The range of small *d* values is flattened by the presence of a minimal scale (controlled by *l*_0_ and *r*), whereas as expected a long power-law like tail appears for large *d*. In this case, there is no other characteristic scales in the model beyond the small scale and $\bar{d}$ as shown by the collapse of the curves for different *p* obtained dividing the *x*-axis by $\bar{d}$ and normalizing the distributions again (figure 3*b*).

The picture changes if *L* and, consequently, the bounding box is finite. The limited Lévy flights are then occurring inside a constrained space and those jumping outside are not considered. This introduces a maximum scale in the *P*(*d*) distributions as can be seen in figure 3*c*, which manifests in a fast (exponential-like) decay for large values of *d*. The curves still maintained the power law-like properties and the possibility of collapse dividing *d* by $\bar{d}$, but in a restricted domain of *d* values (figure 3*d*).

## 3. Results

**Figure 4.**
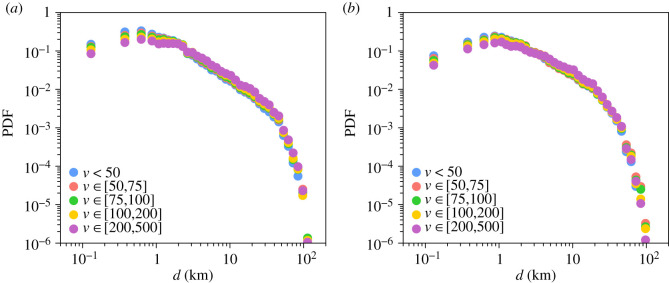
Distribution of the distance travelled according to the amount of money spent in Barcelona (*a*) and Madrid (*b*). Probability density function of the distance between the users’ place of residence and the location of the business in which the transaction occurred according to the amount of money spent *v*. The colour of the curves represents different ranges of amount of money spent.

We start by exploring empirically the relationship between the travel distance *d* and the importance given to its destination *v*. Figure 4 displays the PDF of the distance between the user's home and the location of the business in which the transaction occurred according to the amount of money spent *v* divided into five intervals. Several regimes can be observed. First, the probability to travel a certain distance to make a purchase increases, reaching a maximum between 500 m and 1 km, and, then, the probability starts to decrease, slowly at first, and then more rapidly, exhibiting a power-law-like decay. Finally, after 20–50 km the province boundaries act as a natural cut-off in the distribution (our data are limited to single provinces). The shape of distribution is very similar for each range of amount of money spent. It seems, however, that the distance travelled globally increases with the amount of money spent.

**Figure 5.**
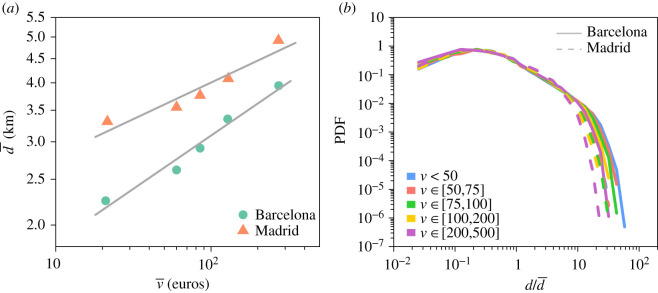
Scaling relationship between amount of money spent and distance travelled. (*a*) Median distance $\bar{d}$ as a function of the median amount of money spent $\bar{v}$ for each range of prices in Barcelona (green dots) and Madrid (orange triangles). (*b*) Probability density function of the distance normalized by the median distance according to the amount of money spent *v* in Barcelona (solid line) and Madrid (dashed line). The colour of the curves represents different ranges of amount of money spent.

Figure 5*a* shows for each range of values *v* the median distance travelled $\bar{d}$ as a function of the median amount of money spent $\bar{v}$. Although the distance travelled is globally higher in Madrid than in Barcelona, the distance travelled increases with the amount of money spent following the scaling relationship $\bar{d}∼{\bar{v}}^{\gamma }$ in both provinces. We obtain a value *γ* = 0.23 ± 0.02 for Barcelona and *γ* = 0.15 ± 0.03 for Madrid estimated with a log-log regression. It is interesting to note that this relation between *d* and *v* seems to be the unique driver of the differences observed between the PDFs in figure 4. Recalling the collapse in the model in figure 3, as can be seen in figure 5*b* a scaling factor depending only on $\bar{d}$ can be used to visually collapse all the PDFs shown in figure 5*b* into a single curve (except for the maximum values constrained by the provinces’ geographical boundaries). This suggests that the mechanisms underlying trip generation are the same for all price ranges and the only difference is a characteristic distance $\bar{d}$, which is a function of the price *v* of the item purchased.

**Figure 6.**
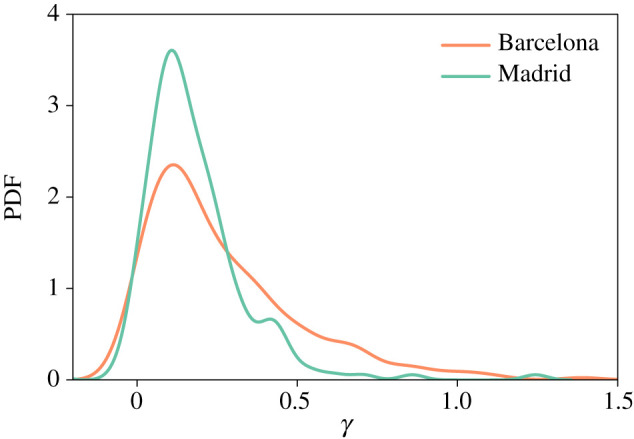
Probability density function of the exponent *γ* estimated with a log-log model for each postcode in the provinces of Barcelona and Madrid.

Nevertheless, we need to verify that this result holds whatever the spatial distributions and types of users and businesses. To ensure that it is the case, we plot in figure 6 the distribution of the exponent *γ* estimated with a log-log model for each postcode in both provinces. The value of *γ* is globally strictly higher than 0, suggesting that the positive correlation between $\bar{d}$ and $\bar{v}$ does not depend of the user’s postcode of residence. Moreover, this positive correlation between the two quantities is independent of the users’ sociodemographic characteristics (gender, age and occupation) and the business categories (see electronic supplementary material for more details).

**Figure 7.**
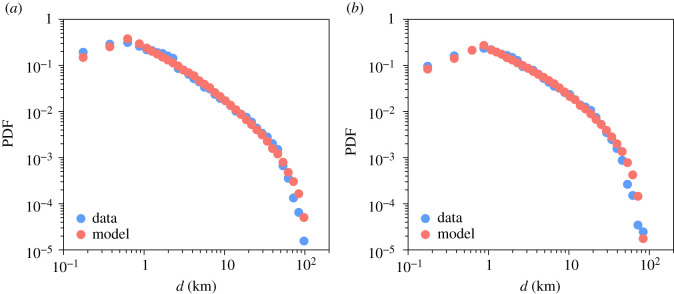
Comparison between data and model. Probability density function of the distance between the user's place of residence and the location of the business in which the transaction occurred (all of the amounts combined) obtained with the data (in blue) and the calibrated model (in red). (*a*) In Barcelona, the best results are obtained with a square of side length *L* = 100 km, a radius *r* equal to 300 m, mobility parameters *l*_0_ = 300 m, *α* = 0.6 and *p* = 0.3. (*b*) In Madrid, the best results are obtained with a square of side length *L* = 75 km, a radius *r* equal to 400 m, mobility parameters *l*_0_ = 400 m, *α* = 0.6 and *p* = 0.25.

We now focus on the results obtained with our model in order to reproduce and explain the relationship between *d* and *v* observed in the data. As described in Material and methods, we first consider the distribution of all the amounts combined in order to calibrate the five parameters. As can be seen in figure 7, the fit is quite good. We obtain similar results in both provinces. The modelling area represented by a square of lateral size *L* is bigger in Barcelona (100 km) than in Madrid (75 km). The parameter *α*, exponent of the Pareto distribution, is equal to 0.6 which is consistent with values obtained in other studies [2–7]. We obtain a value of *p* equal to 0.3 in Barcelona and 0.25 in Madrid, this value, between 0 and 1, has an inverse relation to the energy that people are willing to invest in order to go shopping in both provinces.

**Figure 8.**
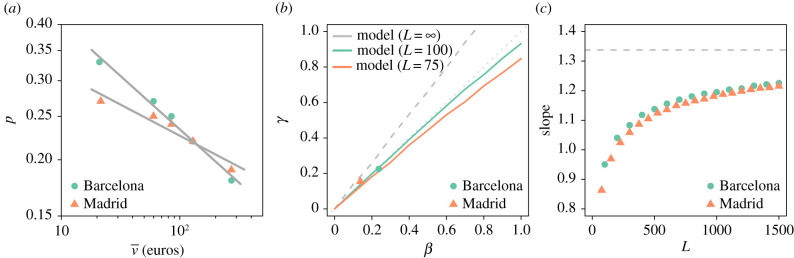
Relationship between *γ* and *β*. (*a*) Probability *p* as a function of the amount of money spent *v* in each bin in Barcelona (green dots) and Madrid (orange triangles). (*b*) Relation between *γ* and *β* obtained with the model and the data for Barcelona (in green) and Madrid (in orange). The lines correspond to the simulations and the symbol to the data (Barcelona in green and Madrid in orange). The grey solid line corresponds to the results obtained with the model with *L* = ∞. (*c*) Evolution of the slope between *γ* and *β* as a function of *L* for Barcelona (green dots) and Madrid (orange triangles).

Finally, we explore the behaviour of *p* according to the median amount of money spent $\bar{v}$. The results obtained are plotted in figure 8*a*. As expected, the value of *p* decreases with increasing *v*, which implies that the distance travelled grows with the price of the item to be purchased. Furthermore, we find that a scaling relation of the type $p∼{\bar{v}}^{-\beta }$ adjusts well to the data. We obtain a value *β* = 0.24 ± 0.01 for Barcelona and *β* = 0.14 ± 0.02 for Madrid estimated with a log-log regression. However, keep in mind that the model does not impose a given relation between *p* and $\bar{v}$, it can be general with different type of data leading to diverse relationships (or exponents if the power-law scaling holds). In our case, both $\bar{d}$ and *p* can be expressed as scaling functions of $\bar{v}$. It is, therefore, important to understand the relation between the direct observable in mobility $\bar{d}$ and our model’s *p*. If the basic displacement distribution had a finite second moment, i.e. the movement was a random walk in 2D, it would have been possible to find analytical approximations for the final distance. However, this task becomes complex with a finite number of steps in a Lévy flight.

Therefore, to analyse the relationship between *γ* and *β*, we assume a relation $p∼{\bar{v}}^{-\beta }$ to generate five *p* values for a given *β* value. We normalize the five *p* values in order to preserve the average value observed in the data (i.e. values displayed in figure 8*a*). We then simulate the five $\bar{d}$ values associated with the *p* values with our model (using the calibrated *L*, *α*, *l*_0_ and *r* values for both provinces). We finally estimate the exponent *γ* from $\bar{d}∼{\bar{v}}^{\gamma }$ and compare the values of *γ* obtained versus those of the corresponding *β*. The results of this exercise are shown in figure 8*b*. The relation is linear and close to the identity (approx. 0.9) but we note a slight difference between the results obtained with the model parameterization used for Barcelona and Madrid. This is mainly due to the size of modelling area *L*. The grey dashed line in figure 8*b* represents the relationship between *γ* and *β* obtained with an infinite modelling area. The effect of increasing the value of *L* on the slope of the linear relationship is also exposed in figure 8*c*. We observe that the slope ranges from 0.85 to 1.1 for values of *L* lower than 500 km, after that it increases slowly until reaching the asymptotic value 1.33. This change in the slope is essentially due to the progressive reduction of the sum of Lévy flights’ truncation. It is worth noting that the slope obtained with values of *L* reflecting an intra-urban mobility scale are very closed to one. An equality between *γ* and *β* is also consistent with the empirical observations made in Barcelona and Madrid (figure 8*b*), suggesting that the probability to stop the journey could be inversely proportional to the median distance travelled ($p∼1/\bar{d}$).

## 4. Discussion

In summary, we introduced a model of individual human mobility patterns able to reproduce and explain the relationship between the travel cost associated with a trip and the importance given to its destination observed in credit card data recorded in the provinces of Barcelona and Madrid in 2011. In particular, we have shown that the distances between place of residence and place of purchase increase with the amount of money spent following a similar scaling relationship in both provinces. The model that we propose is able to reproduce these behaviours and also to mimic the final scaling relation.

Overall, we observed good agreement between the results obtained in Barcelona and Madrid. Both provinces show similar trends in the relationship between the amount of money spent and distance travelled. The exponent values observed in the scaling relationships are of the same order of magnitude in both provinces and the calibrated parameter values obtained with the model are also very similar. More research is needed to elucidate whether the patterns found are common to other countries and cities, and, specially, whether the small differences are related to the diverse city shapes or the geographical structure of the administrative units.

### 4.1. Limitations of the study

The results obtained provide confidence in the robustness of the scaling relationship observed in the data by assessing the effect of the users’ characteristics and business categories on the exponent of the scaling relationship in the two provinces. However, it will be important to evaluate our hypothesis and our model on case studies coming from other countries/continents and on different data sources. A limitation of the study lies in the nature of our data samples, which were spatially constrained by the province boundaries. This forces us to include a bounding box in the model and restricts our capability to reach analytical results. As in [30], we also assumed that every shopping trip starts at home and ends at the place of purchase without considering more complicated case including sequences of purchases. Nevertheless, we replicated the analysis considering, for each user, only days with a unique transaction and we obtained very similar results (see electronic supplementary material, figure S3). Finally, online shopping could be also a handicap for our analysis. Unfortunately, we cannot distinguish online and offline shops in our data. Online shopping has become more relevant with time, its presence was not so strong in the shopping habits of 2011 as it is today, and the retailer must be included in the same province is a strict limitation for most of the online shops. In the online purchases, the distance would not be an important variable and it would not show a clear relation with *v*, given that it is possible to buy equally items of any price.

### 4.2. Concluding remarks

To conclude, this study is a first attempt to quantify the relationship between travel cost associated with a trip and importance given to its destination. The results obtained in this study shed new light on the modelling of human mobility patterns at an individual scale. We are quite aware that trip motivations are very complicated to quantify but we truly believe that it is an important topic. Accurate modelling of daily human mobility patterns in cites is crucial in a wide range of applications. Obtaining better insight into the relationship between trip characteristics and travel motivations would enable better understanding of urban dynamics in order to optimize cities. We hope that more and more (hopefully open) data will be made available in years to come to study the importance of trip destination and its role in the modelling of human mobility patterns.

## Supplementary Material

Supplementary Information

## Data accessibility

All relevant data that support the findings of this study are available in figshare (https://doi.org/10.6084/m9.figshare.12074808.v1).
