# Supplementary material for: On the importance of trip destination for modelling individual human mobility patterns
Source: J R Soc Interface. 2020 Oct 14;17(171):20200673. doi: 10.1098/rsif.2020.0673 (PMC7653376; doi:10.1098/rsif.2020.0673)
Supplement: Supplementary Information [file rsif20200673supp1.pdf]

Supplementary Information for Modeling individual human mobility patterns by travel purpose

Maxime Lenormand[1], Juan Murillo Arias[3], Maxi San Miguel[2], and José J. Ramasco[2]

[1]TETIS, Univ Montpellier, AgroParisTech, Cirad, CNRS, INRAE, Montpellier, France
[2]Instituto de Física Interdisciplinar y Sistemas Complejos IFISC (CSIC-UIB), Campus Universitat de les Illes Balears, E-07122 Palma de Mallorca, Spain
[3]BBVA Data & Analytics, Avenida de Burgos 16D, 28036 Madrid, Spain

1 Data preprocessing

As mentioned in the main text, we analyzed in this study a credit card dataset containing information about 35 million bank card transactions made by credit card users of the Banco Bilbao Vizcaya Argentaria (BBVA) in the province of Barcelona and Madrid in 2011. The dataset used in this study has been already presented and analyzed in [1]. We only applied two filters, one on the average number of transactions per day and another one on the maximum amount of money spent per transaction.

First, we filtered out users with a number of transactions per day higher than three. We determined this threshold by plotting the number of transactions per user as a function of the number of days with at least one transaction. We observe in Figure S1 that most of the users made less than three transactions per day (red line). Only a few users (234 for Barcelona and

613 for Madrid) made more than three transactions per day in 2011 representing less than 0.12% of the users in both case studies.

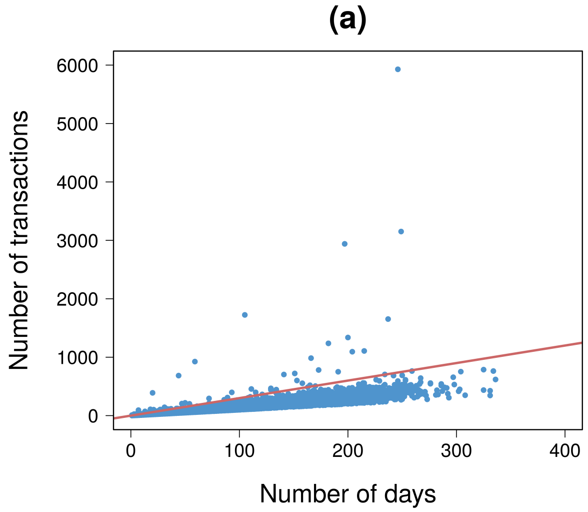

Figure S1: **Number of transactions as a function of the number of days with at least one transaction in 2011 in Barcelona (a) and Madrid (b).** Each blue dot represents a user. The red line represents the threshold of three transactions per day.

Then we removed from the database all the transaction with an amount higher than 500 euros (85,665 for Barcelona and 183,155 for Madrid) representing less than 0.75% of the transactions in both case studies.

2 Effect of the users' characteristics on the exponent γ

As mentioned in the main text the relationship between the median distance $\bar{d}$ and the median amount of money spent $\bar{v}$ can be well-approximated by a log-log function. However, we need to verify that this positive correlation between the two quantities does not depend of the users' sociodemographic characteristics. Each BBVA user is connected with sociodemographic characteristics (gender, age and occupation). For the sake of convenience, we consider five age groups ($]15, 30]$, $]30, 45]$, $]45, 60]$, $]60, 75]$, > 75) and five types of occupations (student, unem-

ployed, employed, homemaker, and retired). The relationship between the distance traveled $\bar{d}$ and the amount of money spent v according to the users' sociodemographic characteristics is displayed in Table S1. Here again, the value of γ is always strictly higher than 0.

Table S1: Relationship between the distance traveled and the amount of money spent according to the users' economic and sociodemographic characteristics.

Category	Median Distance (BCN / MAD)	Slope (BCN / MAD)	R^2 (BCN / MAD)
Total	2.41 / 3.49	0.23 / 0.15	0.97 / 0.91
Men	2.98 / 3.97	0.2 / 0.15	0.99 / 0.87
Women	2.11 / 3.11	0.2 / 0.13	0.93 / 0.84
Age $\in$]15,30]	3.14 / 4.65	0.17 / 0.13	0.99 / 0.91
Age $\in$]30,45]	2.52 / 3.92	0.26 / 0.18	0.98 / 0.86
Age $\in$]45,60]	2.15 / 2.75	0.26 / 0.24	0.96 / 0.96
Age $\in$]60,75]	1.84 / 2.21	0.28 / 0.27	0.99 / 0.99
Age > 75	1.43 / 1.59	0.26 / 0.22	0.92 / 0.99
Student	3.13 / 4.49	0.08 / 0.08	0.92 / 0.87
Unemployed	2.12 / 3.1	0.25 / 0.18	0.95 / 0.95
Employed	2.54 / 3.77	0.23 / 0.15	0.97 / 0.85
Homemaker	1.79 / 2.31	0.25 / 0.2	0.97 / 0.89
Retired	1.69 / 2.02	0.23 / 0.23	0.97 / 0.99

3 Effect of business category on the exponent γ

Finally, we need to verify that the positive correlation between the median distance $\bar{d}$ and the amount of money spent v does not depend on the type of purchases (i.e. business category) in the two provinces. The different business categories and their proportions of associated transactions are available in Table S2. The relationship between the distance traveled and the amount of money spent according to the business category is presented in Table S3. In most of the cases, the value of γ is strictly higher than 0. Note that in some cases, like for the Restaurants business category for example, due to the presence of outlier (Figure 2) no log-log relationship

between $\bar{d}$ and v has been found.

Table S2: Percentage of transaction associated to each of the 20 business categories selected.

Category	Barcelona	Madrid
Supermarket	18.13	16.1
Hypermarket	9.24	11.75
Gas Stations	12.18	11.06
Clothing store chain	4.35	7.23
Restaurants	8.38	6.59
Department store	2.12	5.08
Clothing store chain	5.54	4.58
Pharmacy, optical and orthopedics	4.23	3.78
Retail store	6.32	2.97
Hair and beauty	2.76	2.63
Fast food restaurants and chains	1.02	2.28
Bars and cafe	1.78	1.56
Shoe store	1.57	1.38
Toys and sports articles	1.52	1.37
Electronics, computers and appliances	1.49	1.35
Car dealership, garage and spare parts distributors	1.18	1.1
Bazaar	0.99	1.08
Bookshop, music shop and stationery	1.32	1.02
DIY store	0.75	0.95
Hospitals, clinics, doctors'	0.88	0.87

Table S3: **Relationship between the distance traveled and the amount of money spent according to the business category.**

Category	Median Distance (BCN / MAD)	Slope (BCN / MAD)	R^2 (BCN / MAD)
Supermarket	1.38 / 1.83	0.4 / 0.13	0.85 / 0.98
Hypermarket	2.08 / 2.4	0.37 / 0.25	0.91 / 0.93
Gas Stations	3.22 / 3.71	0.48 / 0.54	0.93 / 0.91
Clothing store chain	4.42 / 4.94	0.02 / -0.01	0.13 / 0.48
Restaurants	5.01 / 5.52	0.03 / -0.04	0.1 / 0.21
Department store	3.68 / 4.82	0.02 / -0.03	0.8 / 0.34
Clothing store chain	2.56 / 4.06	0.14 / 0.07	0.98 / 0.88
Pharmacy	1.52 / 2.01	0.15 / 0.02	0.89 / 0.27
Retail store	1.46 / 1.86	0.24 / 0.18	0.93 / 0.69
Hair and beauty	1.65 / 2.04	0.18 / 0.19	0.89 / 0.92
Fast food restaurants	4.94 / 5.31	-0.15 / -0.11	0.92 / 0.97
Bars and cafe	4.26 / 4.99	-0.07 / -0.11	0.34 / 0.89
Shoe store	2.21 / 3.36	0.21 / 0.12	0.98 / 0.92
Toys and sports articles	3.27 / 5.4	0.24 / 0.09	0.91 / 0.9
Electronics	4.56 / 5.96	0.07 / 0.1	0.93 / 0.89
Car dealership	3.35 / 4.64	-0.08 / -0.04	0.77 / 0.85
Bazaar	2.45 / 3.42	0.22 / 0.1	0.96 / 0.99
Bookshop	2.47 / 3.65	0.07 / -0.14	0.91 / 0.71
DIY store	5.3 / 8.93	0.15 / 0.1	0.98 / 0.92
Hospitals	2.62 / 3.5	0.08 / 0.06	0.9 / 0.45

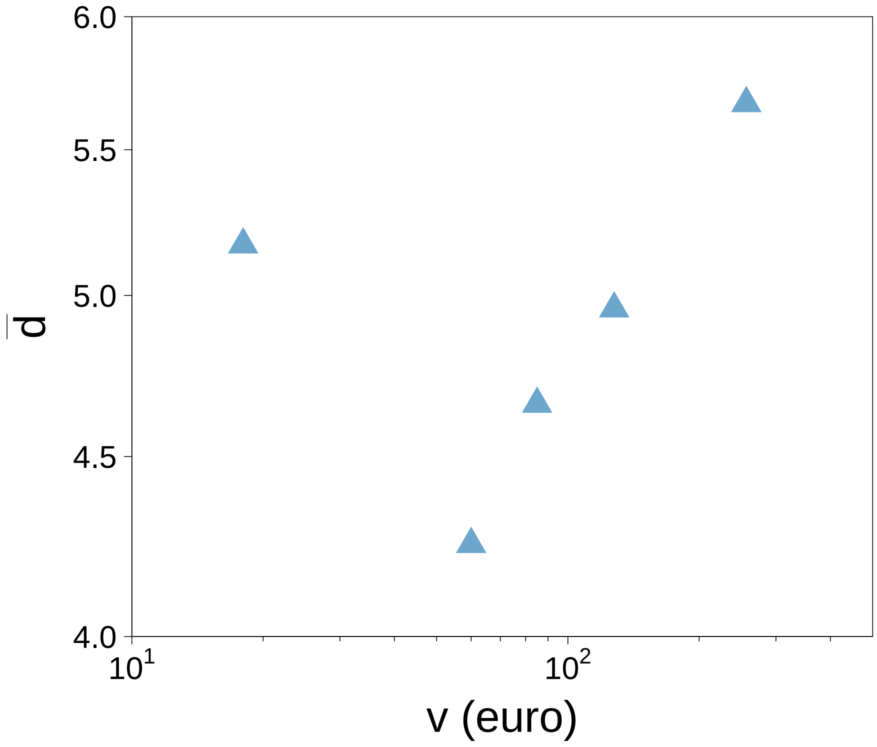

Figure S2: **Median distance $\bar{d}$ as a function of the amount of money spent in each bin for the Restaurants categorie.**

4 Supplementary Figures

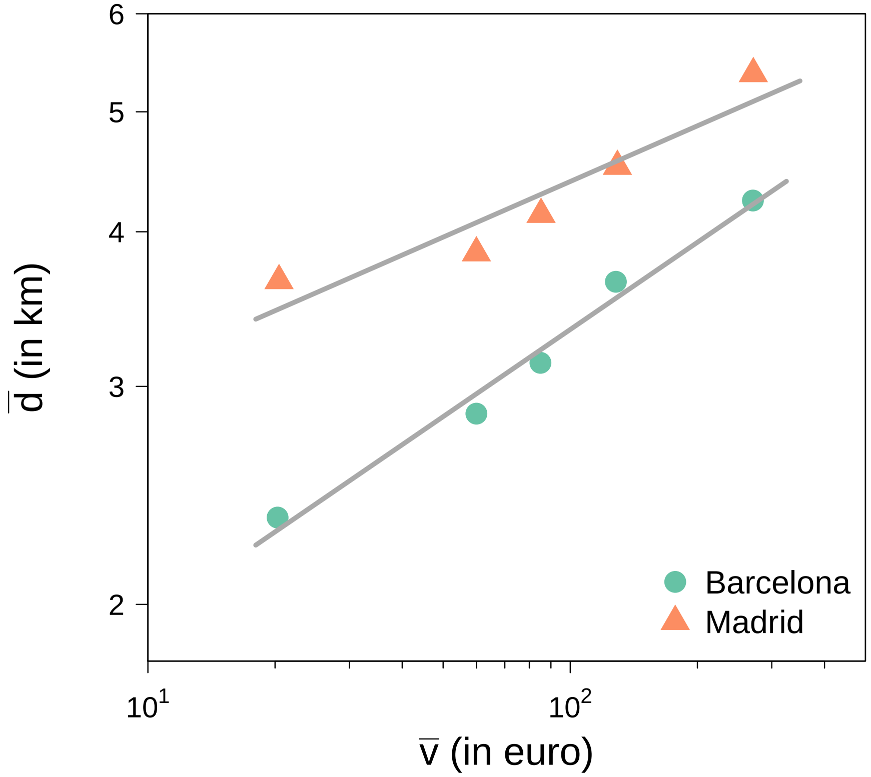

Figure S3: **Scaling relationship between amount of money spent and distance traveled based on daily-unique transaction.** Median distance $\bar{d}$ as a function of the median amount of money spent $\bar{v}$ for each range of prices in Barcelona (green dots) and Madrid (orange triangles). We define a daily-unique transaction as a transaction made by a user during a day where she or he made only one transaction. They represent 39.77% of the transaction in Barcelona and 40.07% in Madrid.

References

[1] M. Lenormand, T. Louail, O. Garcia Cantú, M. Picornell, R. Herranz, M. Barthelemy, M. San Miguel, and J. J. Ramasco. Influence of sociodemographic characteristics on human mobility. *Scientific Reports*, 5:10075, 2015.
